# 

**Published:** 1876-03

**Authors:** 


### ANNOUNCEMENTS FOR THE MONTH.

### MONDAYS.

SOCIETIES.

*Mondays, Mar.* 6 and 20—Chicago Med. Society, regular meetings at Gault House, 8 P.M.

*Mondays, Mar.* 13 and 27 — Chicago Society of Physicians and Surgeons, regular meetings at Grand Pacific, 8 P. M.

CLINICS. *Every Monday.*

At Eye and Ear Infirmary, (Peoria and Adams Sts.) 2 P. M.—Prof. Holmes.

At Chicago College, 2 P. M. *Gynecological*—Prof. Merriman.

At Central Dispensary (239 W. Van Buren St.), 2 P.M., *Gynecological*—Dr. Adolphus; 3 P.M., *Medical*—Dr. Bridge.

At Mercy Hospital, 2 P. M., *Medical*—Prof. Johnson.

LECTURES. *Every Monday.* At Rush College (18th and Arnold Sts.), 9 to 1 o'clock—Drs. Wadsworth, Knox, Sawyer and Jackson. At Chicago College, 8½ to 12½—Profs. Jewell Quine, Merriman, Hyde and Bond; 3 to 6—Profs. Davis and Nelson, Andrews and Quine and Roler.

### TUESDAYS.

SOCIETIES.

*Tuesday, Mar.* 14—Academy of Sciences, regular meeting, 8 P. M. (263 Wabash Av).

CLINICS. *Every Tuesday.*

At County Hospital, 2 P. M., *Medical*—Dr. Bevan; 3 P. M., *Surgical*—Prof. Bogue.

At Chicago College, 2 P. M., *Gynecological*—Prof. Roler.

At Mercy Hospital, 2 P. M., *Medical*—Prof. Hollister.

LECTURES. *Every Tuesday.*

At Rush College, 9 to 12—Drs. Owens, Bridge and Strong; 4—Dr. Danforth. At Chicago College, 8½ to 12½—Profs. Jewell, Isham, Merriman and Bond; 3 to 6—Profs. Davis and Nelson, Andrews and Hatfield, and Byford.

### WEDNESDAYS.

CLINICS. *Every Wednesday.*

At County Hospital, 2 P. M., *Ophthalmological*—Dr. Montgomery; 3 P. M., *Gynecological*—Prof. Fitch.

At Chicago College, 2 P. M., *Gynecological*—Prof. Nelson.

At Mercy Hospital, 2 P. M., *Surgical*—Prof. Andrews.

At Central Dispensary, 2 P. M., *Medical*—Dr. Bridge.

LECTURES. *Every Wednesday.*

At Rush College, 9 to 12, Drs. Wadsworth, Ingals and Knox. At Chicago College, 8½ to 12½—Profs. Hollister, Quine, Merriman and Bond; 3 to 6—Profs. Davis and Curtis, Jones and Quine, and Roler.

### THURSDAYS.

CLINICS. *Every Thursday.*

At Chicago College, 2 P. M., *Gynecological*—Prof. Merriman.

At Central Dispensary, 2 P. M., *Diseases of Chest*—Dr. Ingals.

At Mercy Hospital, 2 P. M., *Medical*—Prof. Johnson.

LECTURES. *Every Thursday.*

At Rush College, 9 to 1—Drs. Hyde, Bridge, Strong and Jackson. At Chicago College, 8½ to 12½—Profs. Hollister, Merriman and Bond; 3 to 6—Profs. Davis and Nelson, Andrews and Hatfield, and Byford.

### FRIDAYS.

SOCIETIES.

*Friday, Mar.* 10—State Microscopical Society of Illinois, regular meeting at the Academy of Sciences, 8 P. M.

CLINICS. *Every Friday.*

At County Hospital, 2 P. M., *Medical*—Prof. Bevan; 3 P. M., *Surgical*—Prof. Bogue.

At Chicago College, 2 P. M., *Gynecological*—Prof. Roler.

At Mercy Hospital, 2 P. M., *On Dis. Eye and Ear*—Prof. Jones.

At Central Dispensary, 2 P. M., *Gynecological*—Dr. Adolphus.

LECTURES. *Every Friday.*

At Rush College, 9 to 1—Drs. Wadsworth, Hayes, Sawyer and Case; 4—Dr. Danforth. At Chicago College, 8½ to 12½—Profs. Hollister, Quine, Merriman and Bond; 3 to 6—Profs. Jones and Curtis, Andrews and Quine, and Roler.

### SATURDAYS.

CLINICS. *Every Saturday.*

At Rush College, 2 P. M., *Surgical*—Prof. Gunn; 3 P. M., *Diseases of the Brain and Nervous System*—Dr. Hay.

At Chicago College, 2 P. M., *Gynecological*—Prof. Nelson; *Surgical*—Prof. Andrews; 3 P. M., *Medical*—Prof. Davis.

LECTURES. *Every Saturday.*

At Rush College, 9 to 1—Drs. Owens, Hay, Strong, and Case. At Chicago College, 8½ to 12½—Profs. Hollister, Quine, Sherman and Hatfield; 3 to 6—Profs. Davis and Nelson, Andrews and Quine, and Byford.

☞ There are six special Clinics daily at the South Side Dispensary (at Chicago College) for students of the Chicago College. A daily Clinic is given at Dispensary of Hospital for Women and Children, 1½ P. M., for students of Woman's Hospital College.

